# A multi-batch design to deliver robust estimates of efficacy and reduce animal use – a syngeneic tumour case study

**DOI:** 10.1038/s41598-020-62509-7

**Published:** 2020-04-10

**Authors:** Natasha A. Karp, Zena Wilson, Eve Stalker, Lorraine Mooney, Stanley E. Lazic, Bairu Zhang, Elizabeth Hardaker

**Affiliations:** 1 Data Sciences & Quantitative Biology, Discovery Sciences, R&D, AstraZeneca, Cambridge, UK; 2 Early Oncology TDE, R&D Oncology, AstraZeneca, Alderley Park, UK; 3 Present Address: Precision Medicine, R&D Oncology, AstraZeneca, Cambridge, UK; 4 Present Address: Preclinical Science Services, Alderley park Limited, Macclesfield, UK; 5 Present Address: Prioris.ai Inc., Ottawa, ON Canada; 6 Early TDE Discovery, R&D Oncology, AstraZeneca, Cambridge, UK

**Keywords:** Phenotypic screening, Cancer, Statistical methods

## Abstract

Phenotypic plasticity, the ability of a living organism to respond to the environment, can lead to conclusions from experiments that are idiosyncratic to a particular environment. The level of environmental responsiveness can result in difficulties in reproducing studies from the same institute with the same standardised environment. Here we present a multi-batch approach to *in-vivo* studies to improve replicability of the results for a defined environment. These multi-batch experiments consist of small independent mini-experiments where the data are combined in an integrated data analysis to appropriately assess the treatment effect after accounting for the structure in the data. We demonstrate the method on two case studies with syngeneic tumour models which are challenging due to high variability both within and between studies. Through simulations and discussions, we explore several data analysis options and the optimum design that balances practical constraints of working with animals versus sensitivity and replicability. Through the increased confidence from the multi-batch design, we reduce the need to replicate the experiment, which can reduce the total number of animals used.

## Introduction

The replicability crisis is a methodological crisis in science where results of many scientific studies are difficult to replicate^1^. This crisis is undermining preclinical research and raises ethical implications over the continued use of animals if the outcomes are questionable^2^. The reasons are multi-faceted, from incentive structure of science to methodological issues in the analysis and design. Research involving living organisms has an additional challenge to replicability in that living organisms are highly responsive to the environment with phenotypic changes with both long- and short-term durations. This ability, described as phenotypic plasticity, is an evolutionary adaptation to ensure optimal fit to the environment and an essential component for survival.

The sensitivity of *in-vivo* experiments and concerns over the inability to replicate studies resulted in a call to increase standardisation and subsequent reporting^3–5^. It was felt that experiments with standardisation of procedures, environmental conditions, genetic background and tested where possible within one day would minimise sources of variation. Therefore, these were proposed as the gold standard approach as this approach would reduce variability, thereby enhance sensitivity and supports reproducibility when the environment is defined precisely. However, in a pivotal study, scientists went to great lengths to standardise the environment and protocols in a characterisation of multiple mice strains within three laboratories with several behavioural screens. Despite this extensive standardisation, they observed disparate results and proposed that interactions between the genotype and local environment led to idiosyncratic phenotypes^6^. The initial reaction was a call to increase the effort to standardise^7^.

The level of responsiveness to the environment, had led to the realisation that it is impossible to standardise sufficiently. Examples of this responsiveness are highlighted by studies such as those by Sorge *et al*. who realised that the sex of the experimenter had a significant impact in the outcome of studies assessing pain^8^. Whilst Turner *et al*. highlighted that environmental noise can have broad systematic effects on both the biology and behaviour of rodents^9^. Likewise, exposure to vibration, such as that from electronic equipment, can induce stress in animals and alter research results^10^. Furthermore, research with data from high throughput phenotyping mice projects has shown that even in highly standardised environments, phenotypes in control mice fluctuate unexpectedly between batches and this was observed in multiple institutes across all quantitative traits studied^11^. These observations have led to the view that *in vivo* studies in highly standardised environments may identify idiosyncratic effects and hence decrease reproducibility which has been described as the standardisation fallacy^2,12–15^.

If you embrace the concept that biological variation is the norm this means, there is no pure treatment effect to estimate. Instead of minimising variation, embracing variability has been proposed as a strategy to ensure conclusions from studies are more representative and thereby improve the generalisability and hence the replicability. This variation should not be introduced randomly but systematically in design. Richter and colleagues^13^ introduced variation through a randomised block design where the blocks differed in a planned way to include variation (systematic heterogenization) and found that heterogeneization on age and housing conditions improved reproducibility of behavioural studies comparing inbred strains of mice. Bodden and colleagues demonstrated that time of day was a suitable heterogenization factor for behaviour studies^16^. The challenge is identifying the heterogenization factor for the phenotype of interest^16^. Kafkafi and colleagues^17^ proposed a statistical solution where they estimated from historic data the susceptibility of a trait to variation due to interaction with the environment and used this to adjust the significance score. This approach however depends on accurately estimating the trait’s susceptibility, and for most traits is currently unavailable. Voelkl and colleagues^15^ explored the potential of multi-laboratory studies through simulations utilising real data looking across 13 interventions in animal models of stroke, myocardial infarction and breast cancer. They found that significant improvement in reproducibility could be achieved by running multi-laboratory studies. Through simulations, they showed that these studies did not require many participating laboratories nor an increase in sample size. Consequently, the authors recommended that a multi-laboratory approach should be the gold standard for late phase confirmatory pre-clinical trials.

However, these late phase confirmatory trials are under-pinned by many individual laboratory studies and we need to explore how we can improve the replicability of these. The variation in control data seen in highly standardised facilities indicates that the rodents are reacting to environmental variation beyond the established known criteria and hence we cannot fully report the environment enabling a true replication. This will be contributing to the finding that 50% of scientists have failed to replicate their own results^1^. We explore the potential of a multi-batch experiment for a laboratory, where the treatment effect is estimated from multiple mini experiments which are run independently. An estimate from multiple studies, will be independent of uncontrolled environmental changesand therefore for the reported meta data should have enhanced replicability. The majority of the literature exploring these reproducibility issues has focused on a genotype differences as the experimental effect of interest. Within this manuscript, we are looking at an effect arising from the exposure to a drug, whilst the context differs the underlying issue (the interaction with the environment) is independent of the experiment effect of interest.

With this more complex design, the data analysis needs to account for the structure in the data to meet the assumptions of the statistical methods being applied. It is unfortunately common^18^ for researcher to fail to account for structure in the data and use inappropriate statistical techniques; such as pooling where the data are combined across the batches and statistical tests which assume independent sampling from a common group are used (such as a Student’s *t* test). In these situations, a true effect could be missed, or an effect incorrectly identified as interesting as batch differences are not accounted for appropriately. The pooling of data could reflect a skill gap in that researchers avoid complex analyses because they do not have the technical knowledge^19^.

We identified three common approaches that could be used to analyse the data accounting for the structure: a meta-analysis, a fixed effect regression approach and a random effect regression approach. A meta-analysis is a well-established method for combining results from multiple scientific studies. As we expect the effect size to vary from study to study, a random effect meta-analysis is appropriate as it will estimate the average treatment effect^20^. A meta-analysis is normally the recommended strategy when only summary data is available. We have explored this strategy, even though the raw data is available, as it is relatively intuitive to understand and implement. In fact, the analysis can be executed with Excel^21^. In these analyses, the mean, standard deviation and number of readings for each group in each study are inputted and the estimate is a weighted mean accounting for the estimated accuracy of each study.

Using a regression approach, we can either treat the factors explaining variation in the model as a fixed or a random effects. The choice depends on the objective and the design of the experiment and can be controversial. Typically, if the investigator is interested in the individual levels of a factor (e.g. male versus female) then the factor is fixed. Whereas, for a random factor the levels of a factor are a sample from a population (e.g. litter 1, litter 2, …., litter n) and the investigator isn’t interested in how individual levels differ but wants to account for variation introduced by them. The fixed effect regression model, treating both batch and treatment as fixed effects, is a common approach to analyse multi-batch data^22,23^. Kafkafi argued that this approach is flawed as it assesses significance by benchmarking the treatment effect only against the within group variation. Instead, they propose a random effect model where the treatment effect is tested against the variability of the batch by treatment interaction plus the within group variability. This approach sets a higher benchmark for significance as an additional source of variation is included. Dixon^24^ argues that the choice is whether you are estimating the treatment effect in a narrow or a broad inference space, where in the broad inference space the conclusion would be related to the entire possible population of environments; whilst in the narrow inference space it would be to the specific levels that were studied. We are looking to estimate a common treatment effect that is not dependent on subtle environmental variation and this could be argued to align to the broad inference space. However, as normally implemented random effect regression models are making more assumptions; such as assuming the random effects are normal distributed. Furthermore, the estimated random effects will likely be unreliable because they are based on only a few batches^24^.

Here we present a case study of a multi-batch study and explore of how to design the experiment and analyse the data. The case study utilises mice with a syngeneic tumour model to study the effect of an immune-oncology (IO) therapy, which is a treatment that recruits the immune system to treat cancer. The increase in clinical success with IO therapies has led to an increase in the use of syngeneic tumour models as these provide an assessment of treatment effects in mice with fully competent immunity and allows investigation of the immune cells that respond to treatment. However, these models are challenging due to aggressive growth rates and deterioration of tumour condition. Preclinical response to IO therapy is variable and typically requires large group sizes and replication of studies. The variability between studies and within treatment groups makes the interpretation challenging.

We explored a new design which starts with a small group size (n = 6–8) but multiple batches (n = 3). To enable quantitative comparison across batches, the rate of growth^25^ was estimated from the tumour growth response data and analysis conducted to estimate the treatment effect. Through simulations and discussions, we explore several data analysis options and the optimum design that balances practical constraints of working with animals versus sensitivity. We explore the impact of the increased confidence from the multi-batch design and argue this reduces the need to replicate the experiment with larger group sizes thus reducing the total number of animals used.

## Results

### Comparison of the estimate of the various analytical approaches

From theoretical considerations and confirmed by simulations (example shown in Supplementary Fig. 1), the distinction between the analytical methods lies not in the estimated difference but in the significance assigned to the differences. These differences will arise through disparities in the degrees of freedom assigned and the estimated variability of the difference.

### Comparison of the false positive rate of the various analytical methods

**Figure 1:**
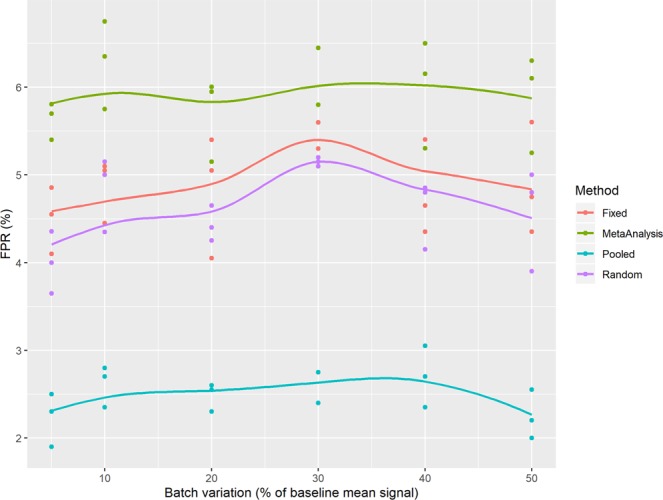
Simulations to explore the false positive rate of the various analytical methods considered. In these simulations, experimental data was constructed to consist of 3 independent batches with a control and treated arm with five animals per group per batch where there was no treatment effect. The growth rate characteristics were based on the characteristics of a MC38 experiment (mean: 0.105, standard deviation: 0.026). Batch variation was assumed to be normally distributed with a mean of 0 and variance varied between 5 and 50% of the baseline growth rate characteristics. This experimental data was processed by the various analytical techniques using a 5% significance threshold. For each scenario, to explore variation in the outcome, this process was repeated 2000 times and the FPR estimated. This simulation process was repeated three times for each scenario explored.

We explored the false positive rate (FPR, equal to the proportion of type 1 errors) as a function of increasing batch variation in simulations when there was no treatment effect between the control and treated group (Fig. 1). For the pooling approach, as the batch variation increased the FPR decreased; which arises from the failure to account for the structure leading to a larger estimate of variance and consequently a reduction in the number of calls made. For the other statistical methods, the FPR was independent of batch variation. The FPR control was excellent for the regression methods as they had a mean FPR with 95% confidence interval that included the expected 5% FPR for the 0.05 confidence threshold. The meta-analysis approach had an elevated FPR returning an average 6.3% (95% CI: 5.7–6.9) but is close to the expectation. The random effect regression and the random effect meta-analysis approach are variants of the same statistical approach, the elevation in the FPR with the random effect meta-analysis likely arises from the use of a z-statistics which is more liberal than the t-statistics used by the mixed model.

### Comparison of the false negative rate of the various analytical approaches

**Figure 2:**
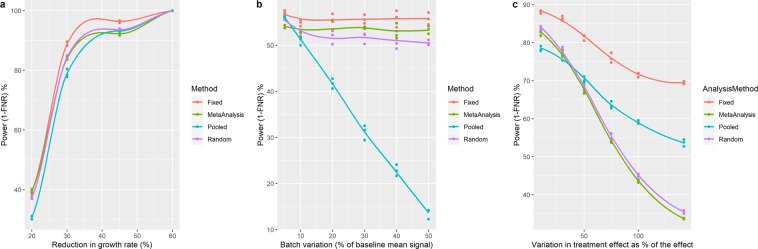
Simulations to compare the false negative rate of the analytical methods considered. (**a**) Sensitivity to detect a consistent treatment effect as the growth rate inhibition increased. (**b**) Sensitivity to detect a consistent 30% growth rate inhibition as the batch variation increased from 5 to 50%. (**c**) Sensitivity to detect a 30% growth rate inhibition as the variation in the treatment effect between batches increased. In these simulations, experimental data was constructed to consist of 3 independent batches with a control and treated arm with five animals per group per batch. The growth rate characteristics were based on the characteristics of a MC38 experiment (mean: 0.105, standard deviation: 0.026). Unless otherwise stated, batch variation was assumed to be normally distributed with a mean of 0 and variance of 20% of the baseline growth rate characteristics. This experimental data was then processed by the various analytical techniques using a 5% significance threshold. For each scenario, to explore variation in the outcome, the process was repeated 2000 times and the FNR estimated. This simulation process was repeated three times for each scenario explored.

From simulations, we explored the false negative rate (FNR, proportion of type II errors) of the various analytical techniques using the baseline line characteristics of a randomly selected MC38 experiment (Fig. 2). Calculating the FNR for various scenarios estimates the sensitivity of the studies as FNR is equivalent to 1- statistical power. The absolute sensitivity will depend on baseline characteristics but allows us to explore the differences between the various analytical approaches. As expected, as the growth rate inhibition increased, the FNR decreased as the statistical power increased. When looking at a treatment effect that is consistent between batches, the sensitivity was similar between the different techniques, however there was a consistent pattern in that the sensitivity was higher with the fixed effect model and lowest with the pooled approach (Fig. 2a). For the three analytical methods, that account for batch, the sensitivity was independent of batch (Fig. 2b). Whereas, for the pooled approach, as batch variation increased the sensitivity decreased. This is arising as the failure to account for the structure in the data results in a high estimate of variance which will decrease the sensitivity. This demonstrates the value of accounting for the structure and supports the statistical advice to not pool the data. As the treatment effect becomes more variable between batches, we see a divergence in the analytical techniques and their sensitivity (Fig. 2c,d). Both the meta-analysis and the random effect model have a greater reduction in the sensitivity as the treatment effect varies. The more conservative behaviour of the random effect model was highlighted by Kafkafi^23^ and arises as it uses both the variance of the batch and the treatment interacting with batch to assess the significance of the treatment effect. If we just consider the FNR for a robust estimate of efficacy we would recommend the random effect regression model or random effect meta-analysis. However, with the elevated FPR for the random effect meta-analysis, where possible the random effect regression model should be used. An advantage of the random effect regression model versus the random effect meta-analysis is the potential to extend the model. For example, a study might need to include a covariate such as body weight to increase sensitivity or address a potential confounding issue.

### Exploration of the optimum design

The optimum design for a model is going to be a function of the effect size of interest, growth rate of the tumour and the variance of the data. Exploring ten sets of vehicle data for the model CT26WT finds that there were no significant differences in the mean growth rate between studies (p = 0.0822; likelihood ratio test comparing a fixed effect model with unequal variance with a null model with unequal variance) (Supplementary Fig. 2). There was a significant difference in the variance between studies (p = 0.0016; likelihood ratio test comparing a fixed effect model with unequal variance with a fixed model with equal variance) with some studies having a 3-fold higher variance than others. Similar results were seen with other models (Supplementary Fig. 2). Consequently, we cannot provide exact recommendation on the design to use. We can provide general observations and a discussion on the issues to consider when selecting the design. The code, used to generate these simulations, has been shared (see section Data availability) and this code could be used with your own measures to assess the optimum design.

Selecting the optimum design is not just a function of animal numbers and statistical power. There are significant practical issues to consider. To avoid mis-dosing errors, account for coprophagia (eating of faeces), and to group house animals to improve their welfare, animals for the same treatment are typically housed in one cage up to a maximum density depending on the cage dimensions and animal size. This does introduce the risk of cage effects to the analysis. This can be minimised by randomly assigning animals to treatment group. Internally, we assign animals to the treatment group by minimisation on body weight which is a valid alternative to randomisation and ensures that the allocation variable will be balanced across the groups^26^. This means systematic differences in terms of litter and original home cage are minimised. The cages are then kept within the same area of the animal house to minimise potential differences. With female animals, a typical maximum cage density is between 5 and 6. This therefore provides a starting point for the group size for a treatment group within a batch.

**Figure 3:**
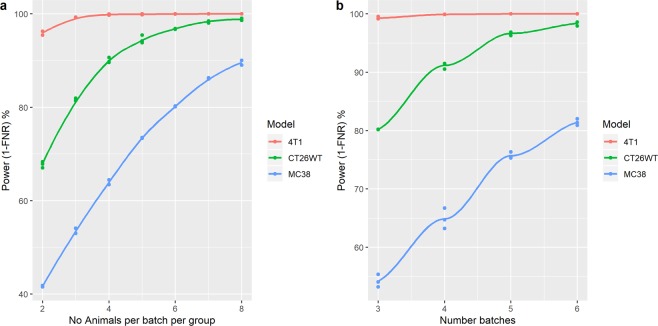
Simulations to explore impact of number the number of animals or batches on the false negative rate. (**a**) Exploration impact of varying the number of animals per group per batch for a three-batch design for three different models on the FNR of the random effect regression model. (**b**) Exploration of impact of varying the number of batches on the FNR when three animals per batch per group with the random effect regression model. In these simulations, experimental data was constructed to consisted of independently sampled batches with a control and treated arm where the treatment effect was normally distributed with a mean effect of a 30% reduction in growth rate with a standard deviation 10% of the mean effect. The growth rate characteristics were the average growth rate for a line and a standard deviation value that encompasses 75% of the values seen. Batch variation was assumed to be normally distributed with a mean of 0 and variance of 20% of the baseline growth rate characteristics. The experimental data generated was processed by the various analytical techniques using a 5% significance threshold. For each scenario, to explore variation in the outcome the process was repeated 2000 times and the FNR estimated. This simulation process was repeated three times for each scenario explored.

From simulations, we explored the impact of varying the number of animals per batch and number of batches on the statistical power (Fig. 3) for the growth rate characteristics of 3 commonly used models. These simulations confirm that power increases both as a function of the number of batches and the number of animals within a batch for all analytical methods studied.

For a set design, the models had very different statistical power. This is both a function of differences in the variability of the models and the average growth rate seen which leads to a different target effect size of interest. MC38 was the most variable model, and this significantly increased the number of animals needed, whilst 4T1 growth rate was found to be quite reproducible between animals. Regardless of power, at a minimum we would recommend 3 batches to be sure the average estimate encompasses three independent environments so the goal of achieving reliable estimates for these conditions are realised. We would also recommend a minimum of three animals per batch per group to allow good estimates within a batch.

As a comparison, we have conducted simulations to explore the number of animals needed if the studies were run in the traditional one batch approach (Supplementary Fig. 3, Table 1). As the variance seen in the growth rate of a model increased, the benefits of embracing the multi-batch design increased. Consider model MC38, a one batch approach would require 28 animals within a single experiment and once replicated would use 84 animals for a two-group comparison. In contrast, the multi-batch design, returns a robust estimate but only requires 36 animals (57% saving).

**Table 1:** Comparison of the number of animals needed for a robust conclusion for variety of models when using either replication of the one-batch approach or the multi-batch design for a study consisting of a control and a treated group.

Model	Multi-batch design			One-batch approach	
	No. batches	No. animals per batch per group.	Total No. animals	Total No. animals/Exp.	Total no. animals after replicated (x3)
4T1	3	3	18	6	18
CT26WT	3	3	18	14	42
MC38	3	6	36	28	84

The number of animals needed were determined from the simulations to achieve an 80% power (20% FNR) at the 0.05 significance level for a 30% change in growth rate using the models’ average growth rate and a standard deviation value that encompasses 75% of the values seen for that model.

### Case studies

**Figure 4:**
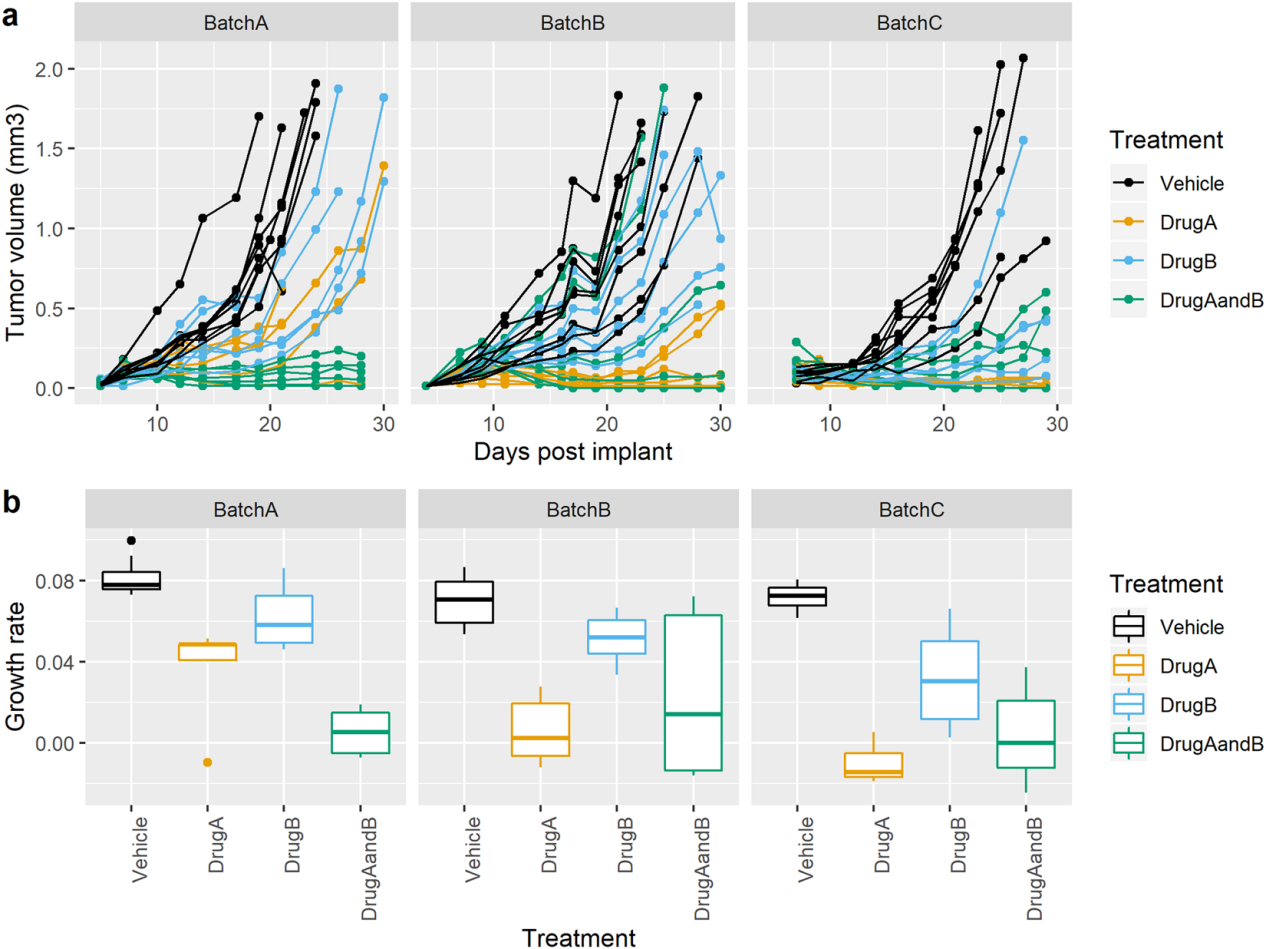
Multi-batch CT26AZ case study 1 growth data. (**a**) Growth profile for each animal coloured by treatment group (**b**) Estimated growth rate for each treatment across the three batches. A boxplot provides 5 summary measures: minimum, first quartile value, mean, third quartile value and maximum. Outliers are shown as individual data points if they are beyond the first/third quartile ± 1.5 *Interquartile range.

**Figure 5:**
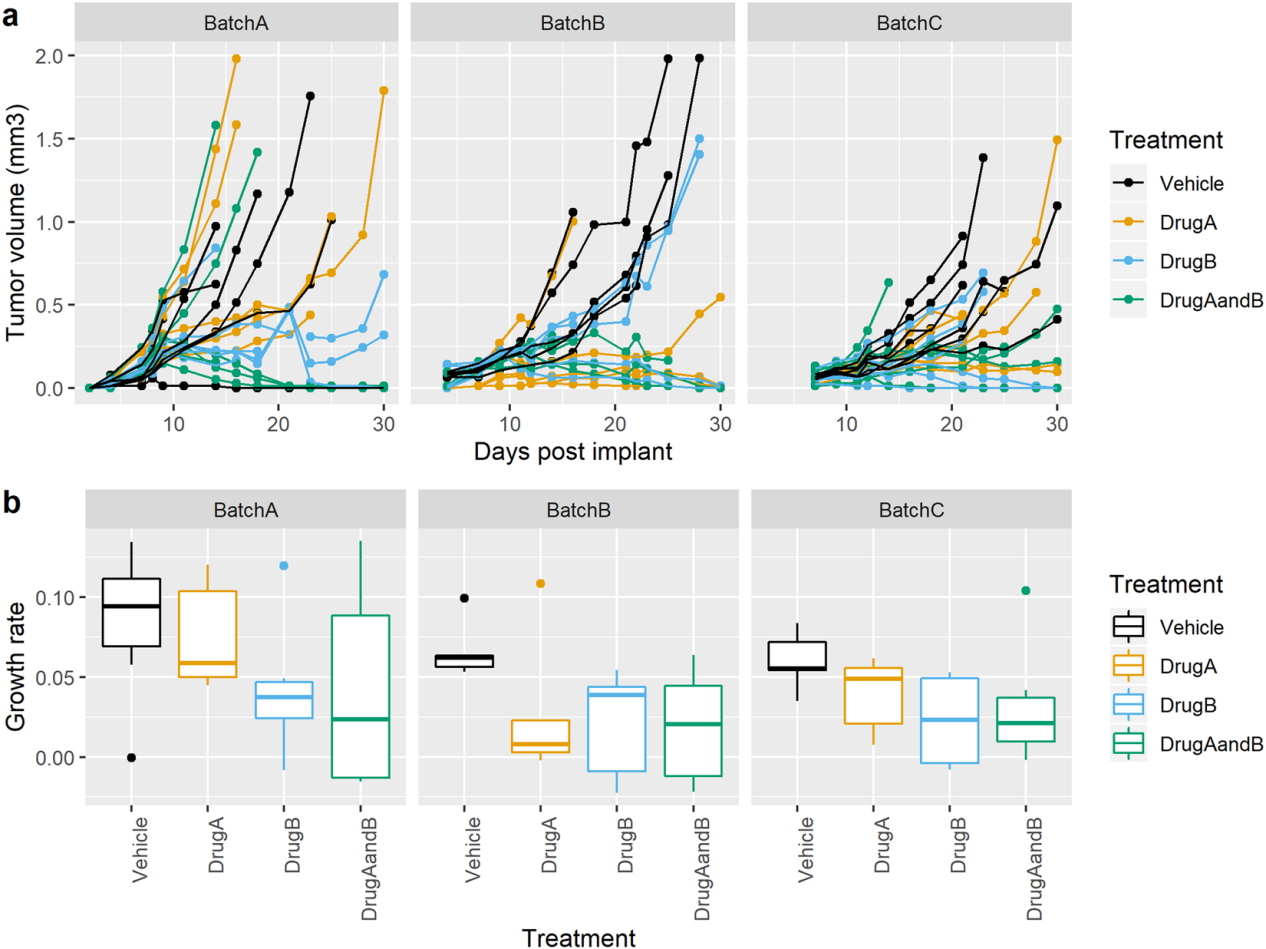
Multi-batch CT26WT case study 2 growth data. (**a**) Growth profile for each animal coloured by treatment group (**b**) Estimated growth rate for each treatment across the three batches. A boxplot provides 5 summary measures: minimum, first quartile value, mean, third quartile value and maximum. Outliers are shown as individual data points if they are beyond the first/third quartile ± 1.5 *Interquartile range.

To demonstrate the multi-batch approach, we ran two studies with a design of 6 animals per treatment group with 3 batches. Each batch was an independent preparation of cells and order of animals from the supplier. Figure 4 and Supplementary Table 1 explore the data for case study 1 on a CT26AZ background. The visualisation of the data, showing the variation within and across the batches is an important component to contextualise the data. The case study highlights the variability in the initial growth rate and variability in the treatment within a batch. However, there is a consistent statistically significant growth rate reduction across the three batches. As this has been seen within our facility on three separate occasion, we have confidence that for our meta data the result is generally replicable with no need for additional replicability attempts. Similar results were seen with case study 2 on a CT26WT background (Fig. 5 and Supplementary Table 2). The results between the 3 analytical techniques that could be used (Fixed, Random or Meta-Analysis) were similar.

## Discussion

The replicability crisis is questioning the efficacy of preclinical research and raising ethical questions about the studies. Here we present a multi-batch design to increase replicability of preclinical *in-vivo* studies for a defined environment. The design is driven by the concept of embracing the replication of studies at the onset. By adopting this concept, we can use a lower n within each batch as the treatment effect is estimated over all the batches. This approach seeks to minimise the potential detection of idiosyncratic findings as subtle environment fluctuations impacts the observed phenotype. This approach would help minimise the experience where scientists themselves have reported failing to replicate their own findings^1^. Thereby, providing a robust platform to move science onwards.

One argument against this strategy is that it is assuming replication of experiments. Replication is considered the cornerstone of science with a role within individual experiments to understand natural variation but also but also in the replication of experiments^27^. In reality, for some areas of science, replication of experiments has become less common. Lack of replication of basic experiments has been highlighted as a major flag for research that cannot be reproduced^28^. With *in vivo* studies, one argument against replication of experiments is ethical over the use of animals. We argue that our proposal is an approach that achieves the goal of embracing replicability but reduces the total number of animals used as the experiment is powered for the concept of combining the data into a single analysis.

The case study, particularly the visualisation across batches, provides a compelling example of the confidence obtained from exploring a treatment effect across three separate experiments where findings were seen with independent batches of animals, cell cultures etc. For the case study explored, looking at the design of immuno-oncology tumor studies, replicability within and between experiments is considered essential because of the high variability in the behaviour. Repeating the experiments with the classic approach of powering each experiment independently is costly; both in terms of finance and in terms of animal usage. Disadvantages of the multi-batch approach are that it takes longer to run a single experiment and the analysis and visualisation is more complex.

The goal behind the multi-batch approach is to assess the treatment effect after the introduction of the uncontrolled variation which will occur between the separate batches. Therefore, we need these batches to be run completely independently. In this case, separate cell preparations, new batch of animals from the supplier etc. To speed up the process, the experimenter could shorten this time window by running the batches independently but concurrently. With a low number of batches, there is then the risk that insufficient variation is introduced to be sure that the estimated effect is independent of subtle environmental variation. A variant would be am and pm testing space, this is then equivalent to the concept of heterogenization by design. Bodden *et al*. found that for behavioural studies estimating the effect from studies blocked by time of day was sufficient^16^. The difficulty with a heterogenization approach to improve replicability is working out what are the critical environmental variables to block on that will be the sources of variation that the phenotype of interest is responsive too.

Within this manuscript, we have explored common data analysis approaches that could be utilised for a multi-batch design. Pooling, which fails to account for structure of the data, had a loss of sensitivity as the variation between batches increased and is not recommended. A random effect regression model and a random effect meta-analysis had very similar characteristics and would be the recommended methods. Both these approaches estimate the average treatment effect but benchmarked the significance by comparing it to the variability within an experiment and variability of the treatment effect. Consequently, if the treatment effect is very variable then the significance of the treatment effect is lower. Utilising these statistical approaches along with the design will return calls with higher confidence in their replicability.

Selecting the optimum design is not just a function of animal numbers, effect size of interest, or variability in the data. There are several practical issues to consider, such as how you house your animals, housing density and how many animals you can process within one batch. Power is increased both by adding animals within a batch and number of batches. Reproducibility will be increased by increasing the number of batches, as the study samples from the variability that can occur for that facility. Where possible, increasing the number of batches should be prioritised. We recommend an absolute minimum of three batches and three animals within a batch even for low variance models. The simulation code used to explore FNR for our experiments has been provided and the functions could be used to explore other scenarios with different values of interest. In the future, there is the potential to future refinement using adaptive design theory where sequential testing could be used to decide if there is sufficient evidence of a potential effect of interest to continue the study and collect all batches.

Replicability issues are affecting all areas of science but is a critical issue for us to address when working with animals as we conduct the harm-benefit analysis when considering our ethical obligations. The 3Rs (Replacement, Reduction and Refinement) act as our guiding principles to frame our ethical use of animals in research. The historic definition of Reduction, with a focus of absolute number of animals in individual experiments, can encourage poor practice that we now understand can leads to replicability issues. This explains the recent development of a contemporary definition of Reduction by the National Centre for the Replacement Refinement and Reduction of Animals in Research (NC3Rs) with a focus on experiments that are robust and replicable^29^. To continue conducting our research using animals, we have to embrace designs that improve replicability.

## Methods

All procedures were conducted in accordance with the United Kingdom Animal (Scientific Procedures) Act 1986 and associated guidelines, approved by institutional ethical review committees (Alderley Park Animal Welfare and Ethical Review Board; Babraham Institute Animal Welfare and Ethical Review Board) and conducted under the authority of the Home Office Project Licences (70/8839, 70/8894 and P0EC1FFDF). All animal facilities have been approved by the United Kingdom Home Office Licensing Authority and meet all current regulations and standards of the United Kingdom.

Animals were housed with an inverse 12 hours day-night cycle in a controlled temperature (20–24 C) and humidity (45 ± 65%) room as directed in the Code of Practice for the Housing and Care of Animals Bred, Supplied or Used for Scientific Purposes. Animals were housed in individually vented cages from Techniplast and were fed Special Diet Services R&M No 1 and watered (water filtered in autoclaved bottles) ad libitum. Within the cage, the bedding material composed of sizzle nest and as environmental enrichment cardboard smart home, cardboard tunnel, Happi Mat and Aspen chew stick. Typically, we housed the animals with up to five animals per cage. The SPF facility has a barrier system and the animals are house on IVC conventional unit (TOTE180-E – Eco-Pure Chips 6).

### Animal details

Immunocompetent female C57BL5 mice were sourced from Charles River, UK and immunocompetent female BALB/cOlaHsd mice were sourced from Envigo, UK. All animals were 5 to 6 weeks old at the time of cell implant. Naïve mice arrive from the external supplier in batches of 5 and are subsequently housed in this same group of 5 when allocated onto study.

### Experimental procedures and study design

#### General design

These studies follow a parallel group design with a vehicle group and multiple treatment arms. Within each study, an individual animal was considered the experimental unit and the primary variable of interest was the growth rate.

Cell lines CT-26.WT and 4 T1 were purchased from American Type Culture Collection (ATCC), whilst MC-38 were from National Cancer Institute (NIH). CT-26.WT (5 × 10^5^ cells/mouse) or MC-38 (1 × 10^7^ cells/mouse) tumor cells were implanted subcutaneously (s.c.) using a cell implant volume of 0.1 mL in the left flank of female Balb/c and C57/BL6 mice, respectively. 4 T1 (1 × 10^4^ cells/mouse) tumor cells were implanted orthotopically in mammary fat pad #8 (o.t.) using a cell implant volume of 0.05 mL in female Balb/c mice. Mice were randomised by cage identification at time of tumour cell implant. Welfare checks were made twice daily, and tumour condition monitored throughout the study. If condition of the animal, tumour, or the humane endpoints were reached the animals were humanely culled. To avoid mis-dosing errors and manage welfare from potential moderate transient welfare effects blinding was not implemented.

To enable the simulations and understand the growth rate behaviour, we retrospectively analysed vehicle group from historic datasets on CT26.WT, MC38 and 4T1 lines.

We piloted the multi-batch design by running two experiments with one control arm and 3 treatment arms. The multi-batch design consisted of 3 batches with 8 animals in control group and 6 animals per treatment group per batch. Compound A and B were formulated in the vehicle Phosphate-buffered saline at the appropriate concentration to dose at 10 mg/kg. Mice were dosed twice weekly i.p. (doses given at the same time in the morning on day of dosing) for the duration of the treatment period.

All animal data was recorded and included in the data analysis.

### Tumour volume measurements

The length and width of the tumours were measured at regular intervals (typically every 3rd day) with digital Vernier callipers. The volumes were estimated using Eq. 1.

1 $$volume=\frac{{(width)}^{2}(length)}{2}$$

### Rate of growth summary measure

The rate of growth for each animal was estimated based on fitting each tumour’s growth curve to an exponential model as described in Eq. 2 ^25^.

where *a* and *b* are parameters that correspond to the log initial volume and growth rate, respectively. The model assumes the error terms are normally distributed.

2 $$lo{g}_{10}(tumor\,volume)=a+b\ast time+error$$

Tumour volume data up until day 30 were fitted with the model and tumour volumes less than 50 mm3 were replaced with a minimum value of 50 mm3.

### Construction of simulated data

Simulated data was constructed to comprise three independent batches with a control and a treated arm. The underlying control population, following historic dataset IO1706, and was assumed to be normally distributed with a mean growth rate of 0.1242 and standard deviation of 0.03916. Batch variation was assumed to contribute to the variation as a random normally distributed variable with a mean of 0 and variance which was defaulted to 20% of the baseline growth rate for most simulations. Exceptions are detailed in the analyses. Depending on the scenario being explored, a treatment effect could be added that was either consistent across the three batches and set as a proportion of the baseline growth rate or alternatively the treatment effect was constructed to vary across batches. When the treatment effect varied, it was assumed to be sampled from a normal distribution and the variation in the treatment effect was set as a proportion of the treatment effect of interest.

### Statistical analysis

#### Fixed effect model

A linear regression model with treatment, batch and an interaction between treatment and batch was fitted to the data (see Eq. 3).

3 $${Y}_{ijk}=\mu +{\alpha }_{i}+{\beta }_{j}+{\gamma }_{ij}+{\varepsilon }_{ijk}$$

where *i* = 1, …, *n*_*T*_, *j* = 1, …, *n*_*B*_ and *k* = 1, …, *n*_*ij*_. In addition, *α*_*i*_ indicates the main effect of the *i*th level of treatment *T*; ${\beta }_{j}$ indicates the main effect of the *j*th level of batch *B*; γ_ij_ is the effect of the interaction term; and the error term ${\varepsilon }_{ijk}$ ∼ *N*(0, *σ*_*e*_^2^). The treatment effect and associated error were estimated across the batches as a marginal mean using the lsmeans package. The significance of the treatment effect was assessed using ANOVA type III sums of squares. For the experimental data, a likelihood ratio test was used to select the variance model (equal variance or variance depending on the treatment) at a 0.05 significance threshold^30^.

#### Random effect model

A mixed linear effect regression model with treatment as a main effect, batch as a random effect and batch by treatment as a random effect was fitted to the data (see Eq. 4).

4 $${Y}_{ijk}=\mu +{\alpha }_{i}+{b}_{j}+{c}_{ij}+{\varepsilon }_{ijk}$$

where *i* = 1, …, *n*_*T*_, *j* = 1, …, *n*_*B*_ and *k* = 1, …, *n*_*ij*_. *α*_*i*_ indicates the main effect of the *i*th level of treatment *T*; *b*_*j*_ represents batch as a random effect where *b*_*j*_ ∼ *N*(0, *σ*_*b*_^2^); c represents batch by treatment interaction as random effect where c_ij_ ∼ *N*(0, *σ*_*c*_^2^) and the error term ${\varepsilon }_{ijk}$ ∼ *N*(0, *σ*_*e*_^2^).

### Random meta-analysis model

A random effect meta-analysis model was fitted to the data using the metafor R package^31^ and was selected as it assumes the effect sizes will differ because they are sampled from a population. First, the mean differences effect size and sampling variance were estimated for each batch. Then, a mixed effect model is used to estimate the treatment effect (Eq. 5).

5 $${T}_{i}=\mu +{e}_{i}+{\varepsilon }_{i}$$

where *T*_*i*_ is the effect size*, μ* is the mean of the underlying effect size distribution, v^2^, is the sampling variance of the effect size, τ^2^ is the variance of the effect size distribution where e_i_ ~ N(0, v_i_^2^) and ε_I_ ~ N(0, τ^2^). The treatment effect and associated error was estimated using the R escalc package to return the estimated raw difference.

### Pooled approach

In the pooled approach, the batch information is ignored and a linear regression model with treatment was fitted to the data (see Eq. 6). For a two-group comparison, this is equivalent to a student’s *t*-Test.

6 $${Y}_{ij}=\mu +{\alpha }_{i}+{\varepsilon }_{ij}$$

where *i* = 1, …, and *n*_*t*_ and j = 1, …, *n*_*ij*_. In addition, *α*_*i*_ indicates the main effect of the *i*th level of treatment *T*; and the error term *ϵ*_*ij*_ ∼ *N*(0, *σ*_*e*_^2^). The treatment effect and associated error was estimated as a marginal mean using the R lsmeans package.

## Supplementary information

Supplementary Information.

## Data Availability

The data scripts used to generate the data and figures have been made available as a Zenodo repository under a creative commons attribution 4.0 license^32^.
