# Supplementary material for: A multi-batch design to deliver robust estimates of efficacy and reduce animal use – a syngeneic tumour case study
Source: Sci Rep. 2020 Apr 10;10:6178. doi: 10.1038/s41598-020-62509-7 (PMC7148295; doi:10.1038/s41598-020-62509-7)
Supplement: Supplementary file 1 — Supplementary Information. [file 41598_2020_62509_MOESM1_ESM.pdf]

A multi-batch design to deliver robust estimates of efficacy and reduce animal use – a syngeneic tumour case study

Natasha A. Karp[1*], Zena Wilson[2], Eve Stalker[2,3], Lorraine Mooney[2,4], Stanley E. Lazic[1,5], Bairu Zhang[1], Elizabeth Hardaker[6]

1: Data Sciences & Quantitative Biology, Discovery Sciences, R&D, AstraZeneca, Cambridge, UK
2: Early Oncology TDE, R&D Oncology, AstraZeneca, Alderley Park, UK
3: Current address: Precision Medicine, R&D Oncology, AstraZeneca, Cambridge, UK
4: Current address: Preclinical Science Services, Alderley park Limited, Macclesfield, UK
5: Current address: Prioris.ai Inc. Ottawa ON, Canada
6: Early TDE Discovery, R&D Oncology, AstraZeneca, Cambridge, UK

Corresponding author*: Natasha A. Karp

Supplementary Figure 1: Bland Altman plot comparing the estimated treatment effect between the different analytical techniques.

a: Fixed effect model versus random effect model. **b**: Meta-Analysis versus random effect model. **c**: Pooled approach versus random effect model. Experimental data was constructed to consist of 3 independent batches with a control and treated arm with five animals per group per batch and where the treatment lead to a consistent 30% reduction in growth rate. The growth rate characteristics were based on the characteristics of a MC38 experiment (mean: 0.105, standard deviation: 0.026). Batch variation was assumed to be normally distributed with a mean of 0 and variance 20% of the baseline growth rate characteristics. This experimental data was then processed by the various analytical techniques. To explore variation in the outcome this process was repeated 300 times.

a.

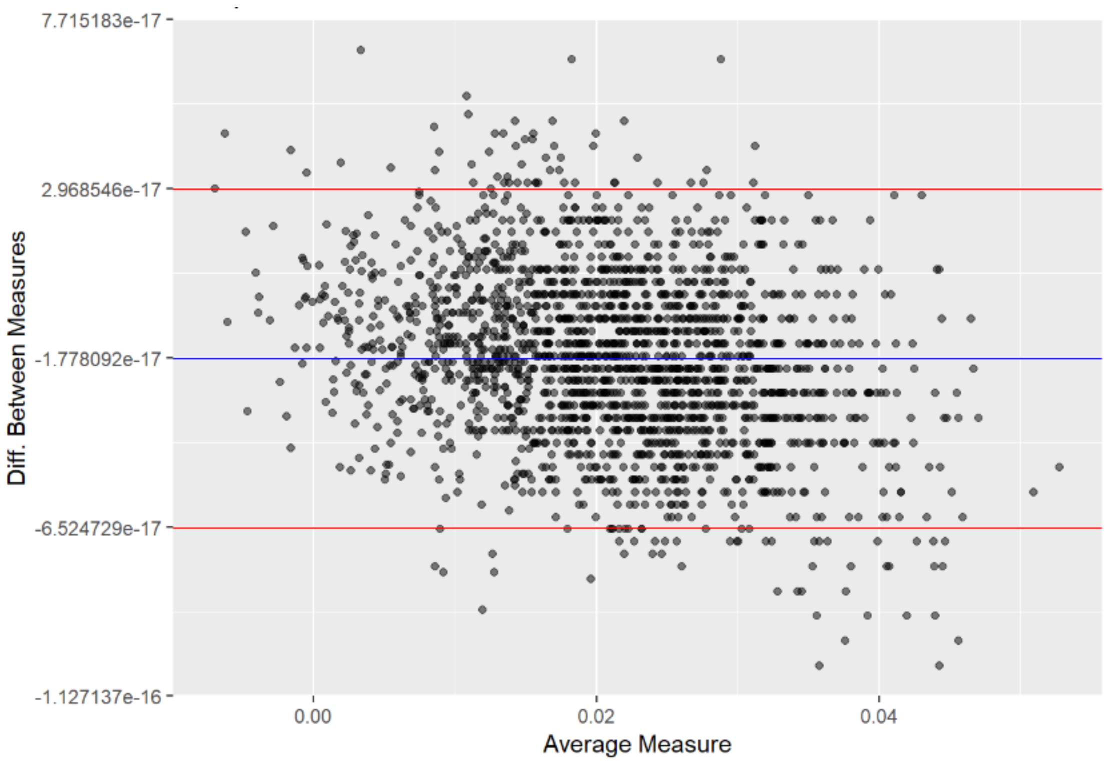

b.

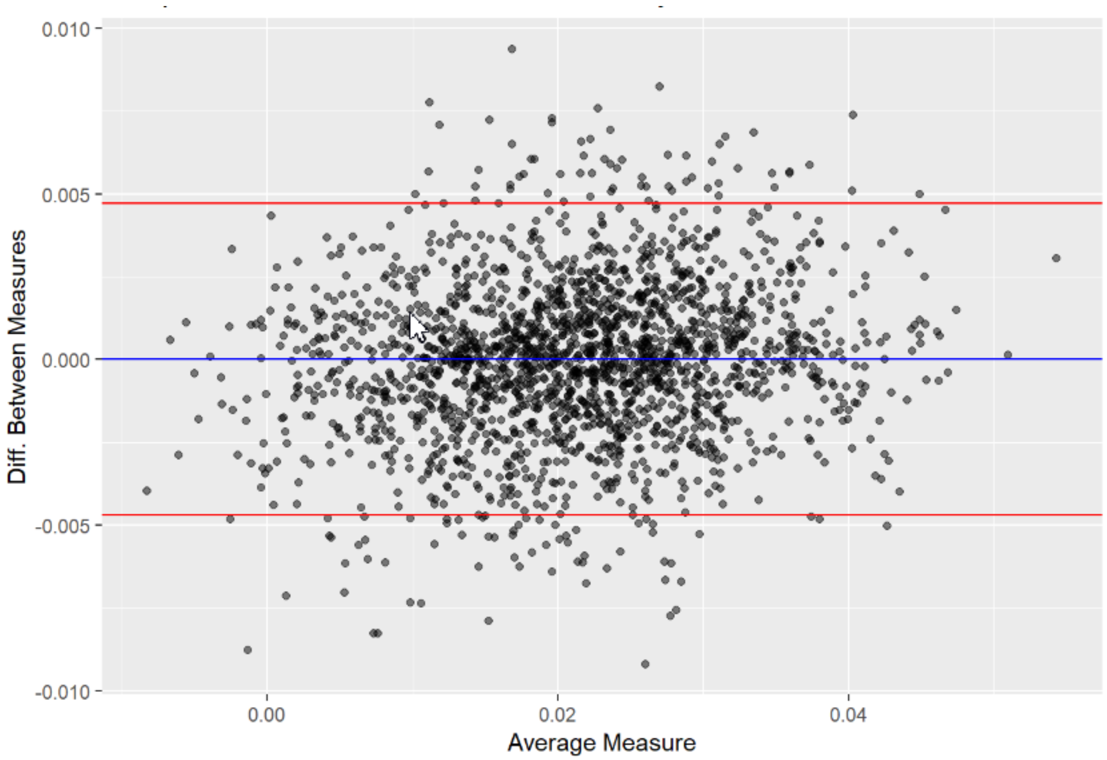
0.010
0.005
0.000
-0.005
-0.010
Diff. Between Measures
0.00
0.02
0.04
Average Measure

c.

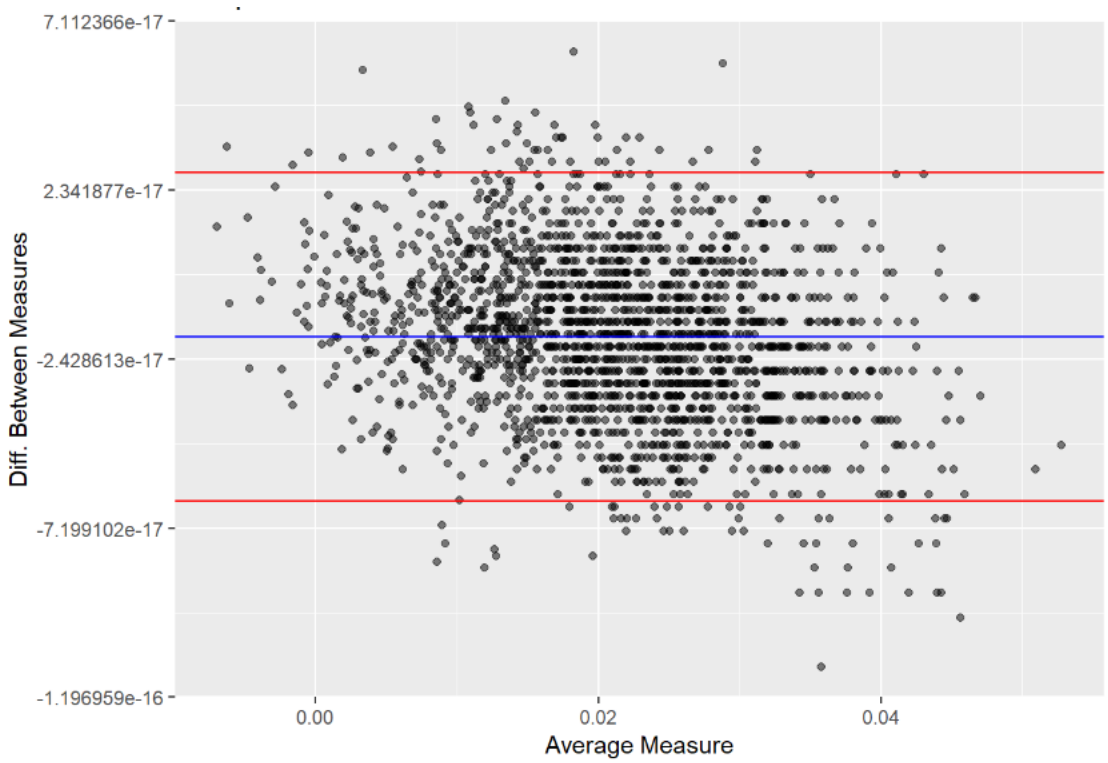
7.112366e-17
2.341877e-17
-2.428613e-17
-7.199102e-17
-1.196959e-16
Diff. Between Measures
0.00
0.02
0.04
Average Measure

Supplementary Figure 2: Exploration of the variability in the growth rate mean and variance seen.

a: The variation in the growth rate for each animal in the CT26WT vehicle group. **b**: The variation in the growth rate for each animal in the MC38 vehicle group. **c**: The variation in the growth rate for each animal in the 4T1 vehicle group. The n per study varied between 8 and 15 animals. A boxplot provides 5 summary measures: minimum, first quartile value, mean, third quartile value and maximum. Outliers are shown as individual data points if they are beyond the first/third quartile ±1.5*interquartile range. The red dotted line indicates the median growth rate across the experiments.

a.

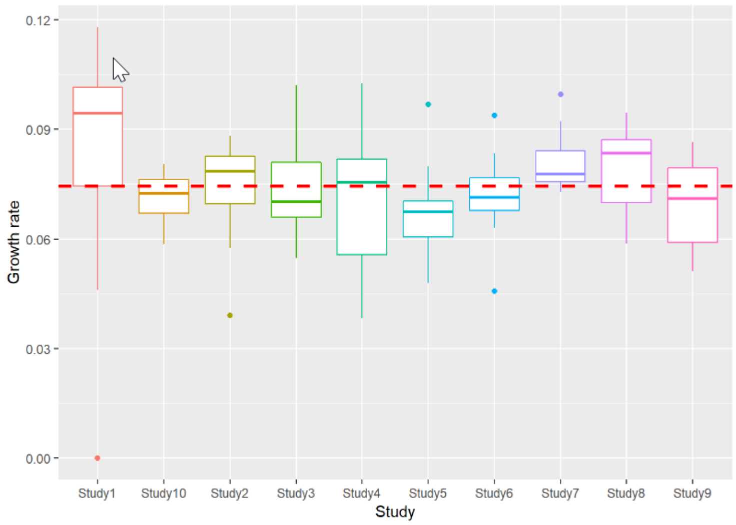

b.

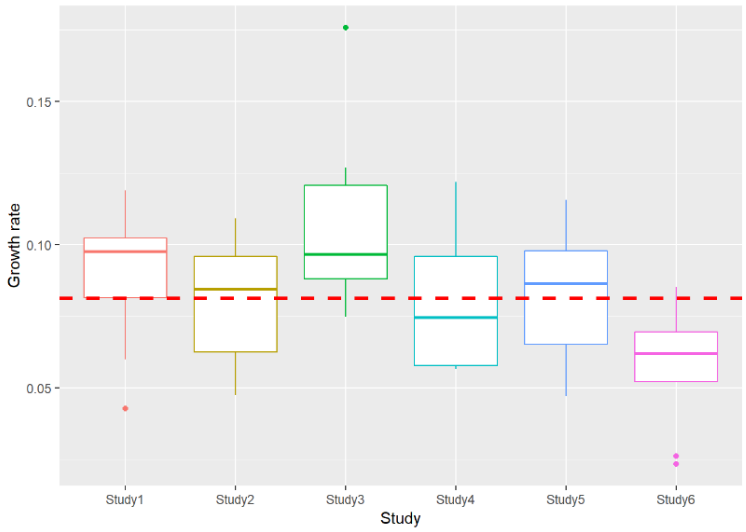

c.

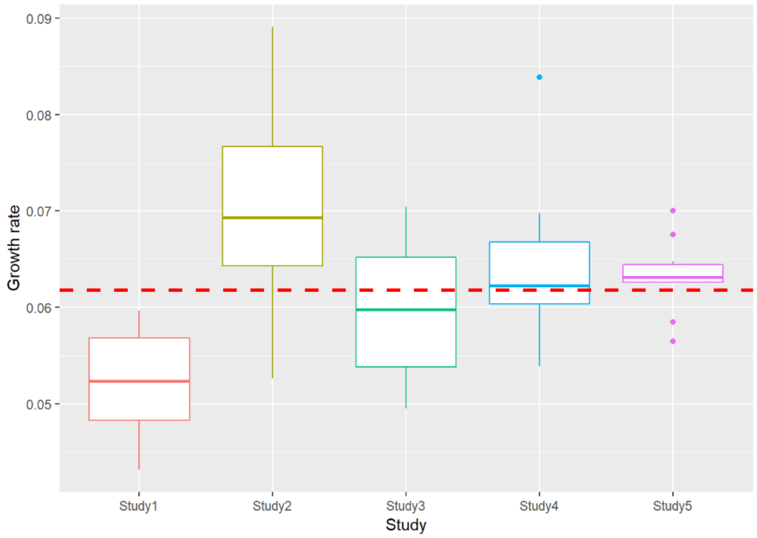
0.09
0.08
0.07
0.06
0.05
Growth rate
Study1
Study2
Study3
Study4
Study5
Study

Supplementary Figure 3: Simulations to estimate the power for a classic one batch design for variety of models.

In these simulations, experimental data was constructed to consisted of a single batch with a control and treated arm with a 30% growth rate inhibition. The number of animals per group was varied. The growth rate characteristics were the average growth rate for a line and a standard deviation value that encompasses 75% of the values seen. The experimental data generated was processed by a regression model with treatment as a fixed effect using a 5% significance threshold. For each scenario, the FNR was estimated by running 2000 simulations. To assess variation in the FNR, the simulation process was repeated three times for each scenario explored.

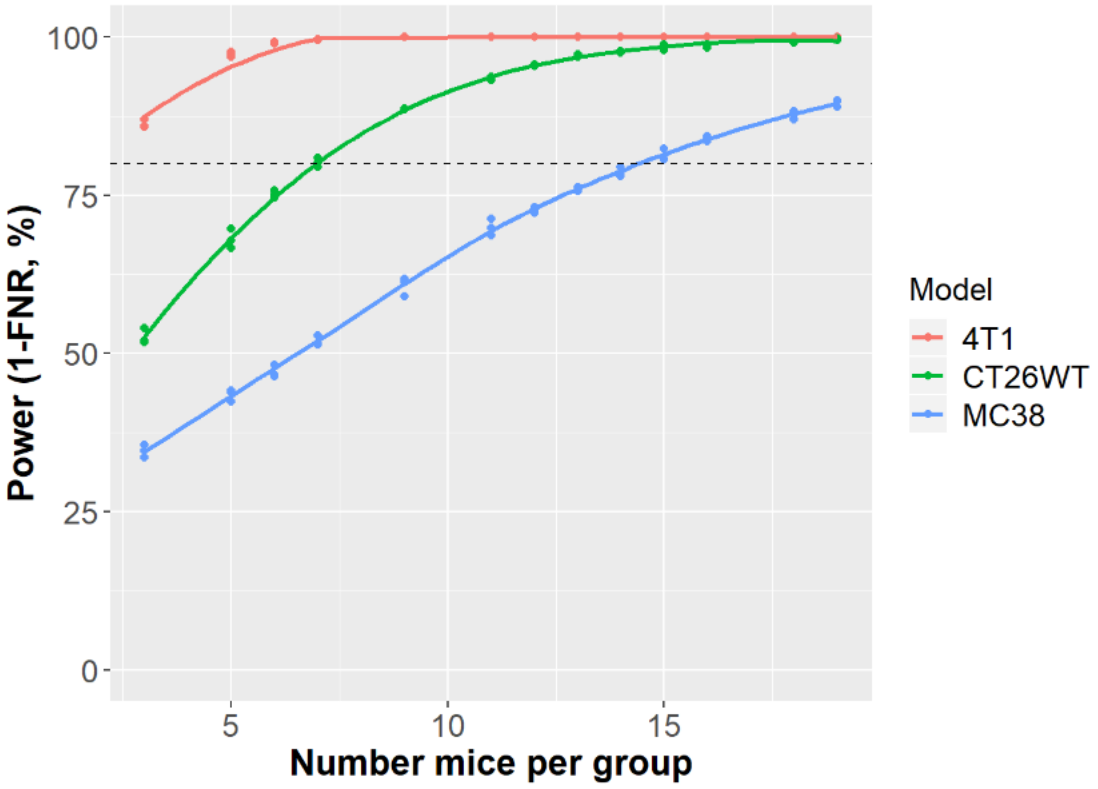

Supplementary Table 1: Multi-batch CT26AZ case study 1 estimated effect and test of significance

Compound	Method	Estimate	SE	P value
DrugA	Fixed	0.0638	0.0043	1.8e-16
	Random	0.0640	0.0102	3.69e-10
	Meta-analysis	0.0661	0.0103	1.45e-10
DrugB	Fixed	0.0260	0.0045	1.32e-6
	Random	0.0260	0.0067	9.83e-5
	Meta-analysis	0.0234	0.0053	1.09e-5
DrugAandB	Fixed	0.0635	0.0070	4.51e-12
	Random	0.0635	0.0069	3.35e-20
	Meta-analysis	0.0708	0.0059	5.27e-33

Supplementary Table 2: Multi-batch CT26WT case study 2 estimated effect and test of significance

Compound	Method	Estimate	SE	P value
DrugA	Fixed	0.0233	0.0111	4.34e-2
	Random	0.0222	0.0108	4.02e-2
	Meta-analysis	0.0224	0.0092	1.48e-2
DrugB	Fixed	0.0419	0.0112	8.05e-4
	Random	0.0417	0.0108	1.12e-4
	Meta-analysis	0.0412	0.0099	2.90e-5
DrugAandB	Fixed	0.0396	0.0136	6.68e-3
	Random	0.0393	0.0131	2.72e-3
	Meta-analysis	0.0382	0.0115	9.27e-4
